# Entrepreneurs and Environmental Sustainability in the Digital Era: Regional and Institutional Perspectives

**DOI:** 10.3390/ijerph17041355

**Published:** 2020-02-20

**Authors:** Qing Ye, Rongting Zhou, Muhammad Azfar Anwar, Ahmad Nabeel Siddiquei, Fahad Asmi

**Affiliations:** 1 Department of Economic Management, College of Information Engineering, FuYang Normal University, FuYang 236041, China; nike007@sina.com; 2 Department of Science and Technology Communication and Policy, University of Science and Technology of China, Hefei 230026, China; rongting@ustc.edu.cn; 3 Bond Business School, Bond University, Robina, QLD 4226, Australia; asiddiqu@bond.edu.au

**Keywords:** green entrepreneur, push–pull–mooring, switching intentions, institutional support, market opportunity

## Abstract

Climate change and environmental degradation have negatively affected the sustainable development of mankind. The “green” concept has been gradually accepted by the public, thereby strongly promoting “green” business forms and social innovation. This study adopts related information and technology knowledge and experience and warm glow (altruistic value) for business initiatives as push factors, market opportunity (MO) and personal innovativeness (PI) in technology as pull factors, and institutional theory (regulatory support and normative support) as mooring factors. These factors are employed to analyze the switching intentions of individuals toward green entrepreneurship, which is a new persuasive psychological model based on Push–Pull–Mooring model (PPM). The survey questionnaires are collected from a total of 1562 respondents through WeChat in mainland China. The study findings present all variables that significantly affect individuals’ switching intentions toward green entrepreneurs. PI exhibits the most significant impact on intention of individuals toward green entrepreneurship, while the interaction between the mooring factor and MO on switching intentions to green entrepreneurship is relatively weak. Finally, the study contributes theoretical and practical implications for increasing intentions toward green entrepreneurship.

## 1. Introduction

Climate change and environmental degradation pose the greatest challenges to mankind [1]. Environmental problems, unfulfilled social needs, and the financial crisis have increasingly influenced the natural ecosystem and human society; thus, the contradiction between economic development and ecological protection has been formed, thereby negatively affecting sustainable development [2]. The business community and establishment have been considered as key networks that are responsible for such problems [3], which elicit the urge to realize new business forms and social innovation [4,5]. 

As the world’s second-largest economic entity, China has been experiencing substantial changes in economic, social, and environmental scopes from the past 40 years, when the economic system has shifted from planned economy to market economy [6]. However, Wu et al. [7] indicated that the remarkable achievements of the economy of China worsened the environmental quality; in particular, the rapid development of the manufacturing industry aggravated environmental resource externalities and pollution. China has faced serious challenges such as resource depletion and environmental degradation [8]. Although the different regions in China are facing various challenges due to different resource endowment, geographical features, economic level and population sizes [9], the common problem of China is the low level of ecological construction including poor environmental protection [10]. Therefore, the government must develop a green economy and increase investment on environmental protection in the future. Li and Lin [8] suggested that the assessment index for local officials in China should add new standards, such as addressing green economic growth and environmental issues.

Chinese policy makers have already been conscious about enhancing the global competitiveness of the economy based on technological development and innovation [11]. Zhang et al. [12] indicated that environmental regulation is an effective approach to address the urgent challenges between environmental protection and economic development in China. The government has implemented a series of appropriate environmental protection measures and policies that can significantly reduce environmental pollution and accelerate economic transition and upgrade. In addition, Zhang et al. [12] realized that people who have experienced serious environmental pollution and non-governmental environmental protection organizations can likewise participate in the decision making for environmental regulation. Fan et al. [13] presented that the development of numerous industries can be stimulated by the environmental protection industry that can be considered as key part of the green economy and can also effectively upgrade the national economy and promote employment.

Most countries have pursued economic development and simultaneously prioritized environmental protection [14]. Business communities, industries, and policymakers have shifted their interests to encourage and develop new sustainable economic forms that can be labelled as “green” [15]. However, owing to the gradual acceptance of the public to the concept of sustainability [16], the rapid growth of the demand for green goods and services has been witnessed [5,17], thereby proving the ecological concern that is widely echoed by the public [18]. Entrepreneurs have been encouraged to operate green entrepreneurship that generates economic and environmental benefits [19]. The green entrepreneurship that connects environment, social, and economic objectives [20] has been recognized as an effective method to establish a sustainable society [21,22,23].

Since the 1990s, green entrepreneurship has been a controversial research field, and its concept could be identified through numerous related terms, namely, eco entrepreneurship, ecopreneurship, environmental entrepreneurship, sustainable entrepreneurship, ecological entrepreneurship, enviro-preneurship, or sustainopreneurship [24]. Vatansever and Arun [23] argued that although certain delicate differences exist among the aforementioned terms, they appear to be unified by a common theme, that is, ecological and social–environmental benefits. Green entrepreneurship is used in the current study because it is a comprehensive concept that fuses ecological entrepreneurship and sustainable development, and it conforms to the “triple bottom line” of the environment, society, and economy.

Compared with traditional entrepreneurship, green entrepreneurship has three unique characteristics, namely, ecological, reliance on green consumers, and reliance on policy support [25]. Green entrepreneurship is regarded as the process that identifies, evaluates, and possesses entrepreneurial opportunities that are based on sustainable, environmentally friendly, and green principles [26]. Green entrepreneurs dedicate to innovate green products and technologies to market so that replace traditional products [27]. Wang and Li [28] indicated that green entrepreneurship must address traditional economic problems and must display concern on social responsibility and environmental issues thus, an individual who exhibits high ecological concerns and social responsibility are willing to operate green entrepreneurship. In summary, green entrepreneurship refers to a new form of entrepreneurship that is obliged to protect the environment and incorporate environmental requirements [27]. Hamdouch and Depret [29] presented that green entrepreneurship is common in most developed countries or regions. However, most green products are relatively expensive than traditional products [30]. Compared with traditional entrepreneurship, green entrepreneurship has a longer return cycle, greater social responsibility, and thus relies more on government encouragement and policy support [31]. To address the challenges faced by green entrepreneurs, the promotion approaches include enhancing awareness regarding environmental concerns in the society, simplifying legal procedures, training qualified “green” workforce, and monitoring the waste of non-green firms [23].

Green entrepreneurship can be categorized into two, namely, established companies that adopted environmental management practices or clean production processes and new enterprises based on natural and ecological resources and thus can be labelled as green entrepreneurship entities [3]. Both require highly green-skilled and experienced professionals. However, developing and developed countries should likewise face the lack of highly green-skilled and experienced professionals, which constitute a constraint on green economy, and such a situation has not significantly improved since 2011 [1]. Developing countries have been challenged by the lack of professionals and a shortage of university graduates in general, particularly those trained in science, technology, engineering, and mathematics (STEM) skills. For example, although the Chinese government has achieved great strides for shifting the economic development of the country into more “green” perspective than before, the lack of a clear green skills mechanism and policy framework has been a major bottleneck in China’s green transition [32].

In the context of increased economic and technological development due to increased economic growth, environmental degradation has been confirmed by researches in social, political, and economic knowledge domains. The scientific consensus on environmental changes, particularly from anthropogenic causes such as high emissions of greenhouse gases, unsustainable consumption patterns, and lifestyle, has raised the environmental concerns among social and business networks. Several industries have incorporated research, which is often supported by institutions, to increase technological innovation, including new sustainable materials, green supply chain, circular economic models, low carbon product procedures, digitalization for waste management, electrification of passenger, and light-duty vehicles, thereby countering these concerns and improving social conditions. Given these technological reforms at the business and industrial levels in an uncertain environment or “changing natural environment” and the market opportunities (MOs) that are created by these environmental concerns, this paper aims to investigate the effects of these technological changes on the intention of an entrepreneur to switch to green entrepreneurship. In particular, this paper aims to determine the behavioral constructs that can help map intentions to switch to green entrepreneurship among recent business students, new entrepreneurial initiators, self-employees, and fresh startup business owners. Moreover, it aims to understand the moderating effect of institutional forces (regulatory and normative beliefs) on the factors that affect green entrepreneurial intentions among youth. The study proposes a new persuasive psychological model that incorporates the push–pull–mooring (PPM) model to analyze the switching intentions toward green entrepreneurship. In the following section, a comprehensive literature review of green entrepreneurship within the context of the proposed theoretical framework is discussed. Thus, the hypotheses are developed, and the methodological aspects are described. The subsequent sections of the paper highlight the interesting findings of the current study that help conceptualize the psychological persuasive behavioral model to fulfil the purpose of the study. 

## 2. Green Entrepreneurship and Push–Pull–Mooring (PPM)

The term “entrepreneur” can be understood as “taking the initiative to bridge,” which originated from France [33]. Entrepreneurship refers to the activities that transform creative ideas and resources into profitable opportunities, which enterprise human identify and exploit new products, processes, or markets [34]. Furthermore, entrepreneurship can produce various outcomes in different forms, thereby promoting economic growth, increasing employment, and addressing environmental problems [35]. Among the various forms, green entrepreneurship that combines environmental, social, and economic objectives has been considered to significantly fulfill the demand for sustainable development. Considerable interests in green entrepreneurship from scholars and the public have been evident since the 1990s, thereby expanding its boundaries. To certain extent, green entrepreneurship can be interchanged with eco entrepreneurship, ecopreneurship, environmental entrepreneurship, sustainable entrepreneurship, and ecological entrepreneurship [24].

Lober [36] indicated that green entrepreneurship is “the creation of new products, services, or organizations to meet environmental market opportunities,” which indicates that companies increase competitive advantage through proactive environmental stances. Gast et al. [26] defined green entrepreneurship as the process that identifies, evaluates, and possesses entrepreneurial opportunities, which are based on sustainable, environmentally friendly, and green principles. Moreover, green entrepreneurship means prioritizing environmental protection and the welfare of the society when entrepreneurs transform conceptual products, technologies, and services into reality [37]. It integrates business entrepreneurship and sustainable development that must take the “triple bottom line” of environment, society, and economy into account [38]. Lotfi et al. [27] presented that green entrepreneurship can be understood as a kind of social activity that aims to protect the natural environment rather than become a mere business. In addition, green consumption has been accepted by an increasing public [14]; thus, increasing opportunities are present for entrepreneurs to operate green entrepreneurship [5]. However, green entrepreneurship must address traditional economic problems and prioritize social responsibility and environmental issues, which means that entrepreneurs who display high ecological concerns, social responsibility, and green skills are willing to operate green entrepreneurship [28].

The previous literature described that researchers have used the theory of planned behavior (TPB) to investigate individuals’ green behavior in different fields [14,39,40,41,42]. Wach and Wojciechowski [43] adopted TPB to investigate the entrepreneurial intentions of students in Poland. Compared with traditional entrepreneurs, green entrepreneurs must prioritize social responsibility and environmental issues during the process of developing conceptual products, technologies, and services [37]. Lotfi et al. [27] indicated that green entrepreneurs are dedicated to innovate green products and technologies to the market. The concept of green entrepreneurship can be extended as a kind of social activity to protect the natural environment [27]. Thus, green entrepreneurship can be defined as a kind of green behavior and a type of switching behavior that shifts the focus of entrepreneurs from economic profits into the concern on social responsibility and environmental issues, particularly as traditional entrepreneurs into green entrepreneurs. 

As a useful conceptual framework for understanding individuals’ switching behavior [44], PPM model has not been used to define green behavior. PPM model originated from the “Laws of Migration,” which was introduced in 1885 [45], and was adopted from push–pull model that was used to explain population migrations [46]. Moon [47] introduced the mooring effect into push–pull model and constructed PPM, which posits that the decision of migrants to move from one geographic area to another is affected by push, pull, and mooring effect factors. Bansal et al. [48] presented the similarity between migration and switching behaviors and adopted PPM to interpret the switching intentions of individuals. Under the framework of PPM, the factors that induce the switching behaviors of individuals can be classified into push, pull, and mooring factors. Push effects, as stressors, refer to the negative factors compelling people away from original location, whereas pull effects refer to positive factors drawing prospective migrants to a certain destination. Mooring effects refer to the supplementary factors that facilitate or hamper migration decisions based on personal or social contexts [49,50]. Recently, PPM has been widely used to interpret switching behavior in different fields, such as user switch between mobile stores [51], consumer switch between physical and mobile stores [52], switching behavior of travelers in the airline industry [44], reaction of customers to cross-channel integration in Omnichannel retailing [53], and voluntary switching intention of users for mobile personal cloud storage services [54].

Green consumption has been increasingly accepted by the public [5,14]. Thus, green entrepreneurship has been widely prioritized on the economic policy agenda by numerous Western governments [3]. A growing number of entrepreneurs have been encouraged to operate green entrepreneurship that connects environment, social and, economic objectives [19,20]. Moreover, compared with the label of traditional entrepreneurs, that of green entrepreneurs includes ecological concerns, social responsibility, and green skills [28]. Green entrepreneurs should face the low scalability and long return of investment periods, which reduce interest from finance providers [55]. Additional costs have also been considered as a competitive disadvantage of green entrepreneurship [56]. A common situation wherein a shortage of highly green-skilled and experienced professionals constrains green entrepreneurship [1]. However, the number of green startups has consistently increased worldwide, despite that green entrepreneurs must equilibrate economic activities, social contexts, and ecological philosophies [57]. The reasons that drive the intentions of entrepreneurs to switch toward green entrepreneurship should be investigated. Escolar-Llamazares et al. [58] indicated that entrepreneurship is a complex human capability that is attributed to confluence of factors. Green entrepreneurship is a considerable multidimensional construct that is determined by a varied set of factors including economic, social, and environmental objectives. Therefore, the current study indicates the factors that drive the switching behavior of entrepreneurs toward green entrepreneurship, which can be categorized as push, pull, and mooring factors.

### 2.1. Proposed Push Factors on Green Entrepreneurship Intentions

#### 2.1.1. Information and Technology (IT) Knowledge and Experience

Dutta et al. [59] indicated that the related knowledge and experience (KnE) of entrepreneurs is a key factor for entrepreneurial intention. Entrepreneurs can identify and gain MOs. Thus, they can improve the understanding of the needs, wants, and demands of the target group of customers through sufficient related KnE. The entire developmental process of an enterprise, particularly a new venture, would significantly be influenced by the decisions and actions of entrepreneurs, while their valuable knowledge promotes entrepreneurial decisions and generates competitive entrepreneurial advantage [60]. Moreover, Chen et al. [60] proved that managerial experience is a key factor for implementing entrepreneurship because it facilitates entrepreneurs to mold entrepreneurial thinking and recognize opportunity. Hörisch et al. [61] found that related knowledge significantly influences the implementation of environmental and sustainability management of enterprises, particularly small- and medium-sized enterprise (SME). Wang et al. [62] indicated that necessary skills or KnEs are the foundation for transforming into an ecologically sound society. By contrast, the lack of related KnE is considered as one of the barriers for implementing green innovation project [63,64]. Klewitz and Hansen [65] also described the key barriers for SME perspective and found that the lack of knowledge about eco-innovation of owner-managers is among such barriers. Conversely, related knowledge about the environment, including general environmental knowledge and eco-label knowledge, exhibits a significant impact on an individual’s attitudes toward the environment [66].

**Hypothesis 1** **(H1).** *Related IT KnE significantly affects green entrepreneurship switching intentions.*

#### 2.1.2. Warm Glow (Altruistic Value) for Business Initiatives

As a multidimensional construct, entrepreneurial intention can be considered a planned behavior that is determined by personal values [58]. Liñán [67] presented that personal values primarily influence entrepreneurial intention. Human values are key driver of pro-environmental behaviors [68]. Numerous researchers have confirmed that altruism, as a personal value, exhibits a significant effect on individual behavior [60,61,62]. [68,69,70]. Altruistic values can be part of personal value structure, which is the guiding principle of individuals that motivates them to contribute to the wellbeing of others or the society as a whole [71]. That is, altruism can be considered as action of individuals to increase the welfare of others and raise self-costs despite the lack of personal gains [72]. Griskevicius et al. [73] indicated that most prosocial behaviors include the characteristics of altruism. Berenguer [74] also presented that altruistic values are antecedent of pro-environmental behavior. A survey showed that leaders who exhibit high levels of altruistic values present benevolent leadership behaviors for promoting the well-being of subordinates rather than control [75]. Furthermore, altruism exhibits an important impact on green behavior [76], and altruistic attitude significantly affects ecologically conscious consumer behavior [77,78,79,80].

Isen [81] indicated that individuals who display moral satisfaction are willing to help others and introduced the term “warm glow” (WG) to describe the emotional experience involved. Individuals may experience psychological benefits from prosocial behavior, that is, a WG effect, which WG motivates pro-social behavior because individuals can experience affective satisfaction [82]. Interestingly, Hartmann et al. [68] found that WG is a stronger driver for pro-social behavior than altruistic value. Altruistic value can hardly be instilled to adults, whereas WG, as an emotional reward, is feasible. Moreover, WG theory explains the specific green behaviors that are related to environmental protection [68]. In conclusion, WG can motive the individual to engage in green entrepreneurship.

**Hypothesis 2** **(H2).** *WG (altruistic value) for business initiatives significantly affects green entrepreneurship switching intentions.*

### 2.2. Proposed Pull Factors on Green Entrepreneurship Intentions

#### 2.2.1. Market Opportunity

MO can be understood as a newly identified need, want, or demand trend for a firm to satisfy customers. As the new kind of business, green enterprises have been developed through environmental problems, unfulfilled social needs, and financial crisis and have been associated with green economy, green market, and green entrepreneurship [5,15]. Western governments have prioritized green entrepreneurship on their economic policy agenda since they adopted green entrepreneurship as a key tool that generates new employment and increases economic growth [3]. Gibbs and O’Neill [15] also indicated that a growing number of policymakers have been dedicated to facilitate and develop new economic forms that are termed as “green.” Therefore, green entrepreneurship, which connects economic and environmental benefits, has been promoted worldwide [19]. However, the success of technological innovations often depends on public opinion [83]. The concept of green consumption has been widely accepted by the public in the past few years [84]. The demand for green productions and services has rapidly grown [17,85] since the concept of sustainability has gradually been accepted by the public [16]. Cai et al. [86] investigated that “green” labels may create MOs for green furniture manufacturers. The green entrepreneurship that intersects among environment, social, and economic objectives [20] has been considered as a key driving force of sustainable economic development [23].

**Hypothesis 3** **(H3).** *MO significantly affects green entrepreneurship switching intentions.*

#### 2.2.2. Personal Innovativeness in Technology

Yi et al. [87] indicated that personal innovativeness (PI) in technology refers to the degree of interest of an individual to operate any new domain-specific information technology or innovation. The innovativeness in technology of an individual can be considered his/her stable trait, which exhibits a significant impact on his/her perception on emerging ITs [88]. Such individuals are willing to try new technologies earlier and shoulder more risks than their peers when they operate new technologies [80]. Stewart et al. [89] assumed that entrepreneurs who exhibit a high level of PI in technology are inclined to innovative in psychological predisposition. Entrepreneurs must act proactively and innovatively given that entrepreneurship is a process that discovers, creates, and exploits opportunities, particularly for the types of entrepreneurship that are labelled as “green” [26]. Dutta et al. [59] indicated that PI in technology is a key driver for the development of entrepreneurial intentions, particularly in emerging technology industries including the green industry. Related innovative technologies facilitate entrepreneur for marking correct decisions in emerging technology industries that exhibit high levels of technological uncertainty [90]. Moreover, green entrepreneurship can be regarded as a result of innovation, and such high innovative enterprises can promptly address the rapidly changing needs of customers and present new solutions for the customers’ problems, thereby creating business value [91]. Girod et al. [92] exposed that PI promotes individuals’ positive attitude toward the target technology, which is more important than a positive attitude toward pro-environmental behavior for the adoption of green consumer technologies.

**Hypothesis 4** **(H4).** *PI in technology significantly affects green entrepreneurship switching intentions.*

### 2.3. Proposed Mooring Factors on Green Entrepreneurship Intentions

Institutional theory can be defined as entrenched practices, technologies, or principles of social interaction [93], which regards the regulatory and normative environments as important principal component [94]. Institutional environments can significantly regulate individuals’ behaviors [95] and organizational actions [96], thereby affecting entrepreneurial activities [31]. Stachowiak and Stryjakiewicz [97] emphasized that institutions have played an important role for regional development. The theoretical boundary of institutional theory has continually expanded and ranged from political science to economics. Institutional theory has been confirmed beneficial to entrepreneurial research [98]. Busenitz et al. [99] indicated that the level and form of entrepreneurial activity is affected by institutional profiles. Gómez-Haro Samuel et al. [100] summarized that institutional environment can be defined as the stable rules, social standards, and cognitive structures in a society, which can guide and constrict business activity, including strong influence on economic behavior, organizational behavior, and entrepreneurship. Arabiyat et al. (2019) [101] also emphasized that the institutional environment can affect the extent of innovative entrepreneurial activities in various countries. Furthermore, Bernardino et al. [102] presented that the intention toward becoming a social entrepreneur is directly and indirectly affected by institutions. Institutional theory explains social entrepreneurship intention in detail [103] with regulative and normative environments as determining factors. Therefore, the current study elucidates institutional environments from the regulatory and normative environment perspective.

#### 2.3.1. Regulatory Environment

The regulatory environment, which is regarded as the guiding principle of social interaction, has been adopted in various fields. Regulatory environment refers to formal and informal rules or incentives that constrain and regularize individual behavior [104]. Valdez and Richardson [105] emphasized that the function of the regulatory environment can be considered as setting rules and establishing rewards or punishments for the society. In literature, numerous scholars have confirmed that the regulatory environment is a crucial factor that constrains and regularizes individuals’ behavior. For example, Wan et al. [106] found that the effectiveness of government policy toward green behavior in Hong Kong is important for the public. Zhang et al. [107] also confirmed that government stimulus facilitates individual switching behavior toward “green” initiatives. Xu et al. [108] presented that the perception of residents on regulatory environment notably impacts their enthusiasm for participating in waste segregation. In addition, the regulatory environment would considerably determine the type of entrepreneurship that enterprises carry out [100]. The perceptions of the regulatory environment significantly affect the intention toward green entrepreneurial behavior [31,109]. Estrin et al. [110] found that green entrepreneurship (social entrepreneurial ventures) easily achieves success under favorable institutional environment. Pathak and Muralidharan [111] also asserted that regulatory environment constructs the foundation for entrepreneurs to achieve success.

#### 2.3.2. Normative Environment

**Figure 1:**
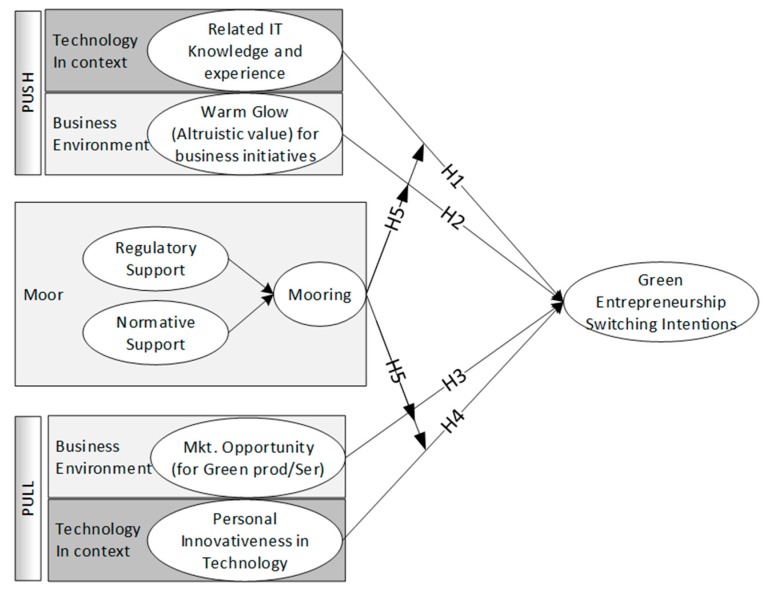
Graphical presentation of the proposed model.

The intention and behavior of an individual is considerably determined by the normative environment, that is, normative pressure perceived by the individual that is potentially attributed to any social actor (i.e., family, friends, neighbors, colleagues and mass media) [112]. The normative environment can also be described as a normative pressure from a combination of injunctive and descriptive norms that follow the perception of acceptable/unacceptable behavior [113]. The normative environment indicates the norms of behavior that restricts individuals’ behavior, which elucidates social value in a particular society [98]. Individuals’ behavior is easily influenced by others, particularly in countries with prevailing collectivist culture [114,115]. Wang et al. [116] investigated that normative environment significantly increases individuals’ willingness toward green behavior. In addition, Gómez-Haro Samuel et al. [100] indicated that the normative environment refers to how the residents of a country value the creative and innovative minds of people and firms, thereby influencing their entrepreneurial orientation. Sambharya and Musteen [117] assumed that the normative institutional environment indicates the direction for an entrepreneur to stipulate the types of entrepreneurial activities that would be admired and supported. The graphical view of the proposed model is shown in Figure 1.

**Hypothesis 5** **(H5).** *Regulatory and normative environments significantly moderate the push and pull factors while defining green entrepreneurial intentions among youth.*

## 3. Methodology

### 3.1. Data Collection Procedure and Sample

We collected the data using survey questionnaires that were gathered through Wenjuanxing, which is the most preferred mode to collect data in the concerned space of population. The survey questionnaires were distributed to a Chinese social networking application (i.e., WeChat). WeChat provides a wide-range data collection approach that helped us reach wide audience and subsequently generalize the research findings. WeChat is one of the popular social networking applications used by the majority of Chinese consumers. Furthermore, researchers suggested using WeChat as a reliable platform to collect data from demographically diversified research respondents [118]. 

The empirical data used in this study were collected from general Chinese consumers through an online survey during the third quarter of 2019. Survey invitation messages that indicate the URL of the questionnaire were distributed to general consumers in China. To encourage participation and create a buzz, we notified that each participant who completed our questionnaires could participate in a lottery draw for an opportunity to win a red envelope ranging from 0.5 RMB to 5 RMB (Abbreviation of the official currency of China, and 1 RMB = 0.16 USD). In particular, more than 2800 potential respondents requested to participate in active or passive manner. A total of 1775 respondents participated. After each response was analyzed, 1562 compete responses were consideration. That is, the response rate of 55.78% was recorded in current research. In terms of appropriate sample size to present the population, the study satisfies the Cochran [119] and Goddon [120] approach to compute for acceptable sample population of an undefined population because the sample size is over the suggested lowest cutoff value. The questionnaires were administered in English. However, given that the respondents were Chinese, we used back-transaction method to carefully evaluate each measurement item for clarity and understanding. Thus, we initially invited three native Chinese who are proficient in reading, writing, and speaking English to assist in translating the English questionnaire to Chinese. We subsequently translated the Chinese questionnaire back to English to remove any semantic discrepancy between the Chinese and the previous English versions. Lastly, we invited 12 participants to examine any equivocal expressions and establish the validity of our questionnaire prior to distributing the final version for data collection. Few items were revised to avoid semantic discrepancy and improved for clarity based on the results.

### 3.2. Measures

This study used well-established and highly reliable scales to measure the study variables. KnE were measured using the five items from the scale of Dutta et al. [59]. A sample item includes “I have the necessary knowledge to adopt technology in business ventures/initiatives.” The scale showed acceptable reliability (α = 0.93). WG was assessed via four-item scale from Hartmann et al. [68]. The scale demonstrated satisfactory reliability (α = 0.93). A sample item involves “Doing eco-friendly business venture/initiatives gives me a pleasant feeling of personal satisfaction.” Regulatory support and normative support individually measured using three items from Urban and Kujinga’s [31] scale. Both measures showed adequate reliabilities. A sample item for regulatory support (α = 0.95) includes “Government organizations assist individuals for starting their initiatives/ventures,” and a sample item for normative support (α = 0.97) involves “Turning new ideas into the initiative is admired in this country.”

MO was evaluated using the three-item scale from Filimona et al. [83]. The scale showed acceptable reliability (α = 0.87) with the following sample item: “Green entrepreneurial initiatives will lead to blue ocean strategy to live in the market.” PI was measured using the four-item scale developed by Dutta et al. [59]. The scale demonstrated acceptable reliability (α = 0.94). A sample item includes “If I heard about new technology, I would look for ways to experiment with it.” Lastly, we measured the dependent variable, namely, green entrepreneurship switching intention using a robust and recent scale from Nguyen, Do, Vu, Dang, and Nguyen [121]. A sample item of this scale includes “I will try my best to start and run my green initiative/ ventures.” The scale demonstrated sufficient reliability (α = 0.93). Table 1 completely lists the items and scale sources. 

## 4. Analysis and Findings

The statistical procedure began with the customary examination of the collected data to check for missing values and normality. Using SPSS, the missing values were analyzed, and the data entry was randomly checked against the original data to ensure accuracy. The degree of skewness and kurtosis was explored for each variable. We employed Armstrong and Overton’s [122] recommendation to use a chi-square test for examining the potential nonresponse bias on all variables by comparing the first and final 25% of all the participants. Results indicated no significant differences, thereby suggesting that nonresponse bias is not a critical threat in our study. We used a two-step analytical approach to test the proposed model. In the first step, we evaluated the reliability and validity of the measurement model. In the second step, we tested our proposed model using structural equation modelling through Smart PLS 3. Table 2 indicates the demographic characteristics of the study sample.

### 4.1. Measurement Model

Confirmatory factor analysis (CFA) was used to measure the convergent and discriminant validities of our construct. CFA is a statistical technique that enables the researcher to test the significance of measurement constructs and the items that measure those constructs [123]. Prior to testing the hypotheses, CFA helps to measure the fit between the theory-based proposed model and the data, which is operationalized by one or more than one goodness-of-fit indices [124]. Table 3 reveals that the Cronbach’s α coefficient and composite reliability of the constructs were above the threshold of 0.7, thereby indicating that all constructs are reliable. Furthermore, the average variance extracted (AVE) values of the constructs were above the recommended level of 0.5. The reflective item loadings that measure each construct were generally above 0.7, thereby suggesting good convergent validity of the scales used in the study. We tested the discriminant validity by comparing the square root of the AVE values of each construct and the correlation coefficients with other constructs. 

The square roots of the AVE were higher than the correlations among the constructs, thereby indicating good discriminant validity. All items were highly and significantly loaded on their respective constructs rather than others, which provide additional evidence of discriminant validity. Table 3 and Table 4 present the discriminant validity statistics that satisfies all the thresholds that were recommended by Fornell and Larcker [125]. Moreover, the alternative way to measure discriminant validity by computing the hetero-trait–mono-trait (HTMT) ratio was also adopted. For all the constructs, the HTMT ratio recorded over the continuum of 0.391 to 0.730. Hence, none of the HTMT ratio bypassed the Henseler’s suggested threshold limit (0.90) [126]. 

### 4.2. Structural Model

We conducted the bootstrapping method with 1562 resamples to test the proposed direct and mediating relationships. Table 5 reports the model fit indices of the proposed structural model and the first and second order CFAs. Hu and Bentler [127] suggested that the model fit values of all CFAs must satisfy the recommended criteria with GFI equal or above 0.95, AGFI equal or above 0.90, TLI equal or above 0.95, IFI equal or above 0.95, NFI equal or above 0.95, and RMSEA at or below 0.08. Table 6 indicates the parameter estimates of the proposed relationships. We report the standardized coefficients and their significance for each hypothesis. All hypotheses were supported at least at the 0.05 significance level.

In terms of direct effect hypotheses, we find a positive and significant direct association between KnE and green entrepreneurship switching intentions (β = 0.199, t = 4.774), thereby accepting Hypothesis 1. This finding was also revealed by the previous pool of researchers [66]. In addition, WG exhibits a positive and significant direct effect on green entrepreneurship switching intentions (β = 0.230), thereby supporting Hypothesis 2 that was consistent with previous studies [68]. Hypothesis 3 is also accepted because of a positive and significant direct relationship between MO and green entrepreneurship switching intentions (β = 0.125), and similar findings were recorded by [86]. Lastly, we found a positive and direct effect of PI on Green Entrepreneurship Switching Intentions (β = 0.273), thereby accepting Hypothesis 4. Similar result was also concluded by [92].

All moderation hypotheses are supported. The interaction between mooring and KnE exhibit a positive and significant effect on green entrepreneurship switching intentions (β = 0.112), which supports Hypothesis 5a. Moreover, we find a positive and significant effect of the interaction between mooring and WG (β = 0.105), thereby accepting Hypothesis 5b. In support of Hypothesis 5c, a positive and significant effect exists on the interaction between mooring and MO on green entrepreneurship switching intentions (β = 0.070). Lastly, we find support for the moderating role of mooring in the relationship between PI and green entrepreneurship switching intentions (β = 0.104), thereby accepting Hypothesis 5d.

## 5. Discussion

The rapid growth of the demands for green goods and services has been witnessed since the concept of sustainability gradually attracted the public. Green entrepreneurship, which has also been considered as a key driving force for sustainable economic development, has concentrated on innovating green products and technologies to replace traditional products. Therefore, green entrepreneurship has been a controversy since the 1990s. The study proposes a new persuasive psychological model based on PPM model to analyze green entrepreneurial switching intentions among recent business students, new entrepreneurial initiators, self-employees, and fresh startup business owners.

### 5.1. Theoretical Implications

The theoretical contributions of the study are specified as follows. First, the current research is the first initiative to emphasize that green entrepreneurship outbalances entrepreneurship. Human beings should face the present greatest dilemmas such as climate change and environmental degradation due to the blind pursuit of economic interests and lost sight for environment and social benefits. Although entrepreneurship exhibits a positive influence on economic growth and job creation, new business forms and social innovation has been required by time. Green entrepreneurship is the process that identifies, evaluates, and possesses entrepreneurial opportunities, which are based on sustainable, environmentally friendly, and green principles [26]. As previously mentioned, green entrepreneurship is a comprehensive concept that integrates ecological entrepreneurship and sustainable development, and it conforms to the “triple bottom line” of environment, society, and economy. Second, the study adopts PPM to investigate green entrepreneurship intention for the first time. PPM model is a useful conceptual framework to understand individuals’ switching behavior [44], but this model has not yet been employed to investigate green behavior. Green entrepreneurship is not only a mere business but is also regarded as a form of prosocial activity [27]. Therefore, the study investigates green entrepreneurial switching intentions among recent business students, new entrepreneurial initiators, self-employees, and fresh startup business owners through PPM model. Third, the study employs WG as the critical factor for operating green entrepreneurship. Altruistic value has been confirmed as a key driver of pro-environmental behavior [76,80]. However, altruistic values can hardly be instilled to adults. As an emotional reward derived from prosocial behavior, WG is more feasible factor than altruistic values. WG can encourage individuals who lack significant altruistic values to engage in pro-environmental behavior because it can work as behavioral antecedent and consequence, thereby strengthening prosocial behavior [68]. Hence, WG is a key element for green entrepreneurship, which is confirmed by the current study.

The fourth contribution of this study is the introduction of the function of PI and its relatedness to green entrepreneurship. Study findings reveal that PI is the most influential factor in the existing model. The previous study defines PI as a type of stable trait among individuals [88], which motivates them to try out new technologies earlier and shoulder more risk than their peers when they operate such technologies [128]. This factor can facilitate an entrepreneur when marking correct decisions in emerging technology industries that exhibit high levels of technological uncertainty [90]. Moreover, green entrepreneurship can be considered a product of innovation, because green entrepreneurship generally aims to innovate green products and technologies to the market and replace traditional products [27]. Therefore, PI increases the possibility of green entrepreneurship. Lastly, the current study confirms that knowledge is theoretically proposed to enhance green entrepreneurial intentions among individuals. In previous studies, the related KnE of entrepreneurs have been identified as key factor for entrepreneurial intention [59,60]. Furthermore, numerous authors have indicated that the lack of related KnE is considered a barrier for implementing green innovation project [63,64]. In summary, related knowledge and PI are integrated to produce sustainable green entrepreneurship.

### 5.2. Practical Implications

The current study provides significant implications for recent business students, new entrepreneurial initiators, self-employees, and fresh startup business owners to enhance green entrepreneurial intentions. Moreover, it can also benefit to policymakers.

First, study results indicate that MO is the weakest variable among the significant factors on green entrepreneurial switching intentions. MO can be understood as new needs, want, or demand trend that a firm can fulfil to enhance the utility of customers. Although the demands of green goods and services have rapidly increased, several barriers to green entrepreneurship remain. To a certain extent, the attraction of the MO of green entrepreneurship is diminished by its barriers and restrictions. One barrier on the main motivations related to the green consumption of consumers is economic benefit (saving money) [129,130]. Most green products are relatively expensive, including green food consumption [30]. Yan Li et al. [131] found that the actual premium price of green products in China is beyond the acceptable price for consumers, which renders green products a less advantageous position than that of traditional products in markets. The government of China should increase its support to green entrepreneurship by adopting interest rates and taxation, deregulation and simplification, financial assistance, information services, and venture capital subsidies. The lack of related KnE is another barrier for implementing green innovation project [63,64]. ILO (2019) [1] reported that the lack of highly green-skilled and experienced professionals is a common situation, particularly in low-income countries. Green entrepreneurship has a longer return cycle and a greater social responsibility than other types of entrepreneurship, because it must address traditional economic problems and prioritizes social responsibility and environmental issues. Moreover, the study presents that mooring factors (institutional theory) mediates the effect of MO, but the effect is the least influential element in the existing model. The reason behind this finding is that reliance on policy support is one characteristic of green entrepreneurship [25]. Although increasing Western governments have prioritized green entrepreneurship on their economic policy agenda [3], many developing regions lack policy frameworks to support “green” initiatives; such a lack of policy framework has been recognized as a major bottleneck in China’s green transition [32]. Therefore, driving green entrepreneurship needs appropriate support from government policies. Second, altruistic value has been confirmed as a key driver of pro-environmental behavior [76,80]. However, WG is a more effective driving factor than altruistic value, because it can encourage individuals who lack altruistic values to engage in pro-environmental behavior [68]. Individuals are willing to incur costs because of positive affective reward from experiencing WG. That is, WG motivates individuals to engage in prosocial behavior, because they can achieve personal happiness from helping others [132]. Interestingly, green entrepreneurship can be considered as a kind of social activity that aims to protect the natural environment [14]. Thus, green entrepreneurs have a high level of ecological concerns and social responsibility [28].

Third, established companies that adopted environmental management practices or clean production processes and new enterprises based on natural and ecological resources can be labeled as green entrepreneurship entities [3]. Both require highly green-skilled and experienced professionals. However, developing and developed countries should likewise face the lack of highly green-skilled and experienced professionals, which constrains green economy [1]. China is particularly challenged by the lack of a comprehensive policy framework for green skills training [32]. 

Fourth, a growing number of Western governments have prioritized green entrepreneurship on their economic policy agenda and have adopted a series of economic and policy measures to encourage green entrepreneurship, such as special interest rates and taxation, venture capital subsidies, information services, and technical assistance [3]. However, many developing regions lack a policy framework to support “green” initiatives. As a representative of developing countries and shifting economies, the entrepreneurial activities of China are challenged to strike a balance between economic benefits and environmental protection [133]. Green entrepreneurship is an effective method to address such challenges [134]. However, the lack of an effective policy framework to support green entrepreneurship is evident in China, despite that the government has experimented in fields such as the photovoltaic, energy-saving lighting, and new energy vehicles industries. Furthermore, the government of China should increase the ecological concerns among consumers to enhance the acceptance of green consumption. Lastly, climate change and environmental degradation pose the greatest challenges to human beings [1]. Most countries pursue economic development and simultaneously prioritize environmental protection [14]. Green entrepreneurship has been recognized as an effective method to establish a sustainable society [22]. The government of China should emphasize green initiatives through green entrepreneurship-related communication and appreciation. The rise of green entrepreneurship yields to a sustainable society for future generations worldwide. Therefore, the national image could be rebranded through green entrepreneurship initiatives.

## 6. Conclusions

The study constructs a new persuasive psychological model that incorporates PPM model to analyze the switching intentions of individuals toward green entrepreneurship. All direct effect hypotheses are supported. Among these hypotheses, PI exhibits the most significant impact on green entrepreneurship switching intentions of individuals, which is followed by WG. A positive and significant effect is also found on the interaction between mooring and KnE, WG, MO, and PI, but they are relatively weaker variables compared with direct effect factors. The effect of the interaction between mooring and MO on green entrepreneurship switching intentions is the weakest among all variables. 

Study findings suggest that the education system should be reformed. In particular, a series of curriculums, including ecological literacy, related knowledge, and PI about “green” initiatives should be incorporated into the formal education system. The entrepreneur curriculum should also be revised by focusing on technical (specific to each occupation) and core (soft) skills (for example environmental awareness and protection), particularly based on STEM skills. In addition, a series of new initiatives that enhance WG effect should be implemented. For instance, green entrepreneurs should be set as good examples and granted extra positive affective rewards, because moral satisfaction encourages individuals to engage in green entrepreneurship. Lastly, the government should offer fiscal subsidies to green products to reduce the price gap with traditional goods. 

The study also exhibits certain limitations. The survey questionnaires were collected from 1562 respondents through WeChat. This application has been considered a reliable platform for collecting data from demographically diversified research respondents in China. However, this methodology can be improved by specifying the demographic characteristics including the origin (urban or rural) or the respondents. We intend to conduct the experimental design by involving fresh graduates with a period of 6 months to 1 year. Moreover, the survey can incorporate qualitative and quantitative analysis, because qualitative survey can enrich the results for future studies. Lastly, a practical implication of this study is the development of a series of curriculums including ecological literacy, related knowledge, and PI about “green” to be incorporated into the formal and informal education system. Therefore, the role of curricula can also be examined in the future to reflect EP intentions.

## Figures and Tables

**Table 1:** Constructs, instruments, and sources.

Construct	Code	Items Description
Knowledge and Experience (KnE)	KnE1	I have the necessary knowledge to adopt technology in business ventures/initiatives.
	KnE2	I have the necessary experience to start/add technology in business ventures/initiatives.
	KnE3	I have the necessary technical knowhow to start/add technology in business ventures/initiatives.
Warm Glow (WG)	WG1	Doing eco-friendly business venture/initiatives gives me a pleasant feeling of personal satisfaction.
	WG2	I feel happy contributing to human wellbeing and the quality of the natural environment by involving or initiating eco-friendly business venture/initiatives.
	WG3	By involving or initiating eco-friendly business venture/initiatives, I feel pleased to do something good for our planet.
	WG4	Participating in eco-friendly business venture/initiatives makes me feel satisfied by giving something back to society and the environment.
Regulatory Support (RS)	RS1	Government organizations assist individuals for starting their initiatives/ventures.
	RS2	Local and national governments support individuals who are starting an initiative/ ventures.
	RS3	The government sponsors organizations that help new initiative/ ventures.
Normative Support (NS)	NS1	Turning new ideas into the initiative is admired in this country.
	NS2	In this country, innovative and creative thinking is viewed as a route to success.
	NS3	Entrepreneurs are admired in this country.
Market Opportunity (MO)	MO1	Green entrepreneurial initiatives lead to blue ocean strategy to live in the market.
	MO2	The advantages of green entrepreneurial initiatives outweigh its disadvantages.
	MO3	Eco-friendly entrepreneurial initiatives are a good way to establish new business venture/initiative.
Personal Innovativeness (PI)	PI1	If I heard about new technology, I would look for ways to experiment with it.
	PI2	Among my peers, I am typically the first to try out new technologies.
	PI3	In general, I am hesitant to try out new technologies (Reverse).
	PI4	I like to experiment with new technologies.
Green Entrepreneurship Switching Intentions (GrB)	GrB1	I will try my best to start and run my green initiative/ ventures.
	GrB2	I decided to establish a company in the future.
	GrB3	My career goal is to become an entrepreneur.

**Table 2:** Surveyed sample profile.

Characteristic	Detail	Frequency	Percentage
Gender	Male	894	57.23
	Female	668	42.77
Age	Under 18	182	11.65
	18–25	473	30.28
	25–35	581	37.20
	Above 35	326	20.87
Prefer Self-employment (instead of employment)	Yes	837	53.58
	No	725	46.41
Believe in Climate Change	Yes	1449	92.76
	No	113	07.23

**Table 3:** Exploring factors and reliability analysis.

Construct	Items	λ	α	CR	AVE
Knowledge and Experience (KnE)	KnE1	0.864	0.934	0.887	0.724
	KnE2	0.855			
	KnE3	0.833			
Warm Glow (WG)	WG1	0.933	0.938	0.956	0.846
	WG2	0.922			
	WG3	0.912			
	WG4	0.912			
Regulatory Support (RS)	RS1	0.820	0.956	0.856	0.665
	RS2	0.816			
	RS3	0.811			
Normative Support (NS)	NS1	0.883	0.977	0.909	0.770
	NS2	0.879			
	NS3	0.871			
Market Opportunity (MO)	MO1	0.818	0.885	0.844	0.643
	MO2	0.798			
	MO3	0.789			
Personal Innovativeness (PI)	PI1	0.804	0.946	0.853	0.592
	PI2	0.778			
	PI3	0.776			
	PI4	0.716			
Green Entrepreneurship Switching Intentions (GrB)	GrB	0.856	0.937	0.883	0.716
	GrB	0.849			
	GrB	0.833			

**Table 4:** Discriminant reliability and correlation analysis.

Construct	M(SD)	VIF	KnE	WG	RS	NS	MO	PI	GrB
KnE	5.242(1.059)	1.613	0.653						
WG	4.962(1.451)	1.460	0.375	0.919					
RS	5.343(1.307)	2.124	0.520	0.455	0.815				
NS	5.173(1.477)	1.748	0.497	0.333	0.492	0.877			
MO	5.265(1.392)	1.711	0.453	0.392	0.473	0.550	0.801		
PI	5.047(1.299)	2.564	0.520	0.536	0.494	0.550	0.558	0.769	
GrB	4.968(1.226)	-	0.465	0.410	0.534	0.459	0.513	0.566	0.846
	By Maximum extraction is 40.876 by Knowledge and Education (as construct)								

**Table 5:** Model fitness indices.

Fitness Indices	Recommended Value	First Order Confirmatory	Second-Order Confirmatory	Proposed Structural Model
Chi-square		710.793	803.187	836.888
Df		184	189	190
Chi-square/df	≤5.0	3.863	4.250	4.405
GFI	0.95	0.963	0.959	0.957
AGFI	0.90	0.945	0.940	0.937
TLI	0.95	0.984	0.982	0.981
IFI	0.95	0.989	0.987	0.986
NFI	0.95	0.985	0.983	0.982
CFI	0.95	0.988	0.987	0.986
RMSEA	≤0.08	0.043	0.046	0.047
Recommended values followed the thresholds recommended by Hu and Bentler (1999).				

**Table 6:** Parameter estimation for proposed model.

Sr.	Description	Beta (β) and Significance (ρ)	Result
H1	KnE→GrB	0.199 ***	Supported
H2	WG→GrB	0.230 ***	Supported
H3	MO→GrB	0.125 ***	Supported
H4	PI→GrB	0.273 ***	Supported
H5(a)	KnG *M→GrB	0.112 ***	Supported
H5(b)	WG *M→GrB	0.105 ***	Supported
H5(c)	MO *M→GrB	0.070 ***	Supported
H5(d)	PI *M→GrB	0.104 ***	Supported

Note: *** = Significance level of 0.001
